# Fractionated stereotactic radiotherapy of benign skull-base tumors: a dosimetric comparison of volumetric modulated arc therapy with Rapidarc® versus non-coplanar dynamic arcs

**DOI:** 10.1186/s13014-016-0632-8

**Published:** 2016-04-18

**Authors:** Fanny Martin, Florian Magnier, Lucie Berger, Jessica Miroir, Emmanuel Chautard, Pierre Verrelle, Michel Lapeyre, Julian Biau

**Affiliations:** Department of Radiotherapy, Centre Jean Perrin, 63011 Clermont-Ferrand, France; Department of Medical Physics, Centre Jean Perrin, 63011 Clermont-Ferrand, France; Clermont Auvergne University, EA7283 CREaT, 63011 Clermont-Ferrand, France

**Keywords:** Benign skull base tumors, Fractionated stereotactic radiotherapy, Novalis, Volumetric modulated arc therapy, Dynamic arc therapy

## Abstract

**Background:**

Benign tumors of the skull base are a challenge when delivering radiotherapy. An appropriate choice of radiation technique may significantly improve the patient’s outcomes. Our study aimed to compare the dosimetric results of fractionated stereotactic radiotherapy between non-coplanar dynamic arcs and coplanar volumetric modulated arctherapy (Rapidarc®).

**Methods:**

Thirteen patients treated with Novalis TX® were analysed: six vestibular schwannomas, four pituitary adenomas and three meningioma. Two treatment plans were created for each case: dynamic arcs (4–5 non coplanar arcs) and Rapidarc® (2 coplanar arcs). All tumors were >3 cm and accessible to both techniques. Patients had a stereotactic facemask (Brainlab) and were daily repositioned by Exactrac®. GTV and CTV were contoured according to tumor type. A 1-mm margin was added to the CTV to obtain PTV. Radiation doses were 52.2–54 Gy, using 1.8 Gy per fraction. Treatment time was faster with Rapidarc®.

**Results:**

The mean PTV V95 % was 98.8 for Rapidarc® and 95.9 % for DA (*p* = 0.09). Homogeneity index was better with Rapidarc®: 0.06 vs. 0.09 (*p* = 0.01). Higher conformity index values were obtained with Rapidarc®: 75.2 vs. 67.9 % (*p* = 0.04). The volume of healthy brain that received a high dose (V90 %) was 0.7 % using Rapidarc® vs. 1.4 % with dynamic arcs (*p* = 0.05). Rapidarc® and dynamic arcs gave, respectively, a mean D40 % of 10.5 vs. 18.1 Gy (*p* = 0.005) for the hippocampus and a Dmean of 25.4 vs. 35.3 Gy (*p* = 0.008) for the ipsilateral cochlea. Low-dose delivery with Rapidarc® and dynamic arcs were, respectively, 184 vs. 166 cm^3^ for V20 Gy (*p* = 0.14) and 1265 vs. 1056 cm^3^ for V5 Gy (*p* = 0.67).

**Conclusions:**

Fractionated stereotactic radiotherapy using Rapidarc® for large benign tumors of the skull base provided target volume coverage that was at least equal to that of dynamics arcs, with better conformity and homogeneity and faster treatment time. Rapidarc® also offered better sparing of the ipsilateral cochlea and hippocampus. Low-dose delivery were similar between both techniques.

## Background

Tumors of the skull base, such as meningioma, pituitary adenoma, and acoustic neuroma, are a challenge when delivering radiotherapy because of their close proximity to organs at risk (OARs) [1–3]. A sufficient dose has to be delivered to the target volume while also sparing the numerous OARs, such as the brainstem, chiasm, optic nerves, cochleas, hippocampus, and healthy brain.

Benign tumors grow without invading the surrounding structures and have a low tendency to undergo metastasis, thus leading to a long life expectancy [1, 4–8]. Generally no prophylactic irradiation of larger regional volumes is needed for these tumors due to their minimal invasiveness. Thus, highly sophisticated techniques are needed to avoid damage and to achieve objectives. Indeed, fractionated stereotactic radiotherapy (FSRT) can be of interest to treat this kind of tumors [9–12]. An appropriate choice of radiation technique may significantly improve the patient’s quality of life after treatment. Non-coplanar multiple dynamic arcs have been often used to achieve high conformity while sparing OARs [2, 13–17]. More recently, volumetric modulated arc therapy (VMAT) has been widely developed. VMAT aims to achieve several objectives at once: (i) to improve sparing of OARs and healthy tissue; (ii) to maintain or improve the same degree of target coverage; and (iii) to reduce treatment time.

The purpose of our study was to compare the dosimetric impact between non-coplanar dynamic arcs (DA) and VMAT using Rapidarc® (RA; Varian Medical Systems, Palo Alto, CA, USA) in terms of sparing of OARs, target coverage, and low-dose delivery to treat benign skull-based tumors using FSRT.

## Methods

### Patients

Thirteen consecutive patients treated in our institution with FSRT during the year 2013 for benign skull-base tumors, sized >3 cm in diameter, were included in this study. There were three meningiomas, four pituitary adenomas, and six acoustic neuromas. Eight patients had undergone previous surgery and were being irradiated for recurrence. All patients were treated with Novalis TX® (Varian Medical Systems, Palo Alto, CA, USA and Brainlab, Feldkirchen, Germany) using a non-invasive thermoplastic mask plus a localizer box (Brainlab, Feldkirchen, Germany). Positioning of the patients was performed using a ExacTrac® stereoscopic X-ray system (Brainlab, Feldkirchen, Germany) and a robotic couch with 6° of freedom. Treatments were permitted when the setup error was <0.7 mm translation and 0.7° of rotation [10–12, 18].

A planning CT was acquired of 1.25-mm thickness and was matched with the dosimetric MRI sequences of interest using Iplan® TPS, version 4.1 (Brainlab, Feldkirchen, Germany). The gross tumor volume (GTV) was defined using contrast-enhanced MRI and/or other sequences of interest. For non-operated patients, the clinical target volume (CTV) corresponded to the GTV. For operated patients, the CTV corresponded to the GTV +/− the tumor bed. The CTV was then expanded symmetrically by 1 mm in all dimensions to create the planning target volume (PTV). This margin was determined to account for setup errors of the patient, motion within the thermoplastic frameless mask, plus repositioning system Exactrac® [10–12, 18].

A conventional prescription was given: i.e., 52.2 Gy to 11 patients and 54 Gy to 2 patients (1.8 Gy/fraction). The following OARs were contoured: lenses, eyeballs, optic nerves, chiasm, pituitary gland, brainstem, spinal cord, cerebellum, cochlea, hippocampus and healthy brain (which corresponded to the brain minus PTV). The bilateral hippocampus were considered as a single organ and contoured on a T1-weighted MRI axial sequence using the RTOG 0933 hippocampal atlas [19]. Following Gondi et al., a hippocampal avoidance region was generated by expanding the hippocampal contour by 5-mm volumetrically margin [19, 20].

Considering that the geometric uncertainty in the radiotherapy process was <1 mm, according to our defined stereotactic conditions, we did not draw margins around the OARs when to produce planning OAR volumes (PRVs) [21].

### Treatment plans

Two treatments plans were created for each case: DA and RA. Experienced physicists independently created each plan. Plans were optimized using High Definition MLC 120 (HD MLC). This had a spatial resolution of 2.5 mm at the isocenter for the central 8 cm and of 5 mm in the outer 2 × 7 cm region.

The planning process consisted of two steps. The first step was to obtain that at least 95 % of the PTV received 95 % of the prescribed dose. The second step was to optimize the OARs sparing without compromising PTV coverage. For the OARs, the following dose constraints were used: for chiasm, optic nerves and brainstem, maximum doses (D2%) <54 Gy; for spinal cord, D2% <45 Gy; for cochleas, a mean dose (Dmean) <30 Gy and a D2% <40 Gy (when feasible) and for lenses, a D2% <2 Gy [19, 20, 22, 23].

#### Non-coplanar dynamic arcs (DA)

All DA plans were created with arcs of 6-MV photons. Five non-coplanar arcs were used for twelve patients and four for one patient. The final calculations were performed using Iplan® TPS, version 4.1, using a pencil-beam algorithm with a spatial resolution of 2.5 mm. This formed a conformal treatment where the leaf positions were adjusted every 10° using Beam Eye View. The collimator was set at zero for all plans.

#### Coplanar VMAT Rapidarc® (RA)

All the RA plans were created with two full coplanar arcs for 6-MV photons. The first arc was planned in a clockwise direction and the second in a counter clockwise direction. For all the plans, the collimator was rotated at 30° for the first arc and at 330° for the second arc to reduce the tongue-and-groove effect. The maximum dose rate was set at 600 MU/min. Couch parameters were also added to the plan to account for attenuation of the mega-voltage beams. Final calculations were performed using AAA algorithm on Eclipse® version 10 (Varian Medical Systems, Palo Alto, CA, USA) [24]. The arc optimization algorithm, Progressive Resolution Optimizer used in Rapidarc®, optimized leaf position, dose rate, and gantry speed. Arc amplitudes were contained between 60 and 120°. RA plans followed the International Commission on Radiation Units and Measurements Report 83 recommendations [25].

#### Quality control

After approval from the radiotherapist and physicist, quality controls were done to compare the calculated and measured radiation doses. For RA, we used a 3D comparison with a Delta 4 PT® system (ScandiDos, Uppsala, Sweden). Plans were accepted when >95 % of the local gamma index values were above the criterion of 3 % and 3 mm [26]. For DA, the plans were verified using a punctual ionization chamber measurement (PinPoint® ionization chamber, PTW, Freiburg, Germany) positioned in a Lucy® 3D QA Phantom (Standard Imaging, Middleton, USA) and were accepted when the gap between the calculated and measured radiation dose was <2 %.

### Analyses

The plans analyses were based on dose–volume histogram (DVH) data. For OARs D2% and Dmean were noted according to OAR type. For the hippocampus, we added D40% [19, 20, 27]. For PTV, the parameters of V90%, V95%, and V107% were also picked up as well as D50%, D95%, D2%, D98% and Dmean. Vx% is the volume of the structure receiving a dose ≥ x%. Dx% is the minimum dose that receive x% of the structure volume.

We also calculated two indexes for the PTV: a conformity index (CI) and a homogeneity index (HI).

The CI was defined as follows:

*CI* = (*V*95 % ∩ *VPTV*)^2^/(*V*95 * *VPTV*) [25, 28]

Where V95 % was a volume within the 95 % isodose and VPTV was the volume of the PTV. The higher values of CI indicated better PTV conformity.

HI was defined as follows:

$$ HI = \left[D2\%-D98\%\right]/ Dmean $$

where D2 % was the dose delivered to 2 % of the PTV volume, D98 % was the dose delivered to 98 % of the PTV volume, and Dmean was the mean dose to the PTV. Small values of HI indicated more homogeneous irradiation of the PTV.

Statistical analysis was performed using R v2.15.1 (http://www.cran.r-project.org). To compare the doses for the different modalities, non-parametric Wilcoxon tests for paired samples were used. If the associated *p*-value was less than the significance level (α = 0.05), it was assumed that there was a statistically significant difference between the compared data sets.

## Results

### Target coverage (Fig. 1a)

**Fig. 1:**
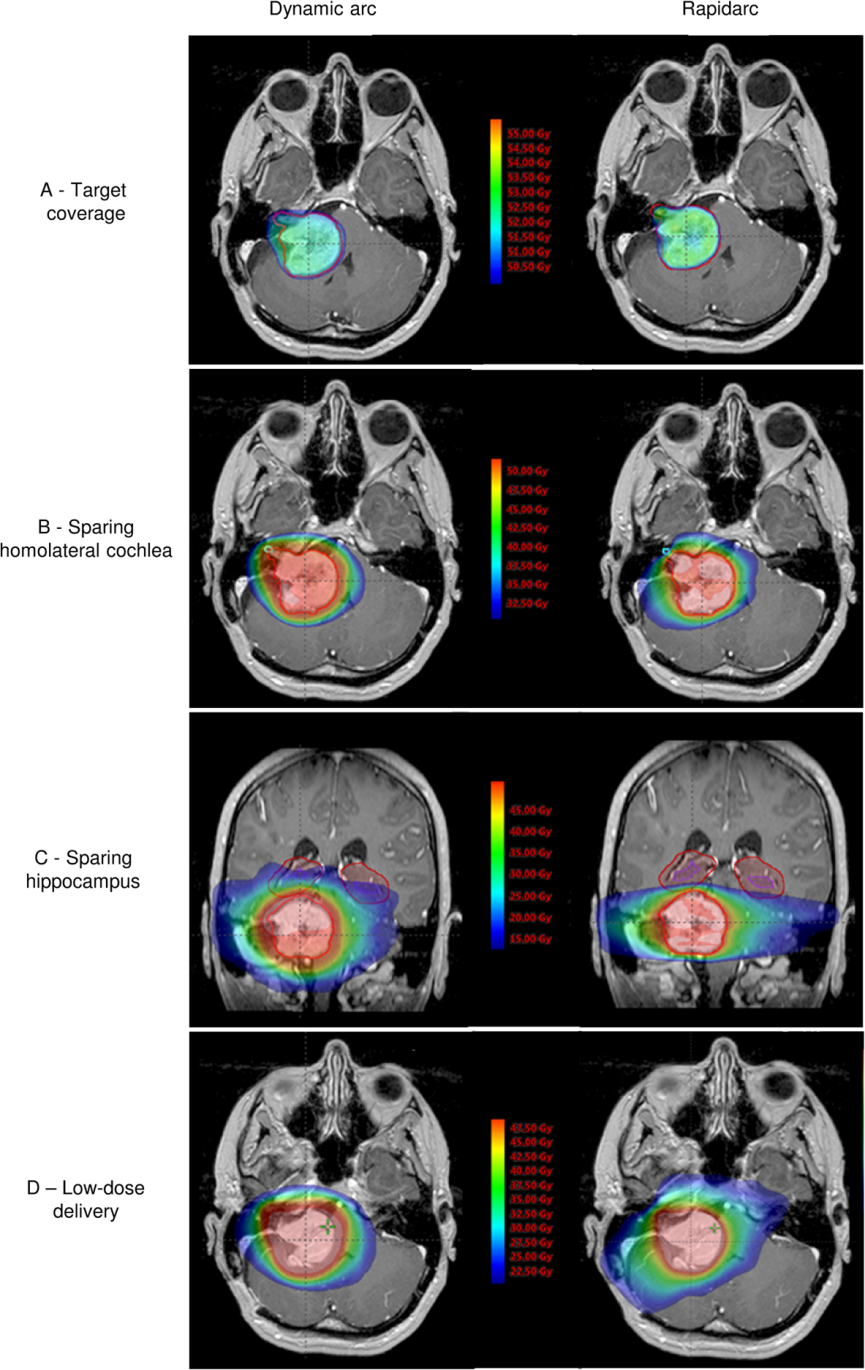
Example of the dose distribution of a patient with a large skull base tumor with non-coplanar dynamic arcs (left) and VMAT (RapidArc®) (right). **a**) Comparison for target coverage. **b**) Comparison for sparing ipsilateral cochlea. **c**) Comparison for sparing hippocampus. **d**) Comparison for low-dose irradiation

The mean volume of CTV was 23.0 cm^3^ (range: 3.1–69.5). The mean volume of a PTV was 29.7 cm^3^ (range: 4.7–135.5). Table 1 summarizes the results of DVH analyses for target coverage. All DA and RA plans achieved 95 % isodose coverage for at least 95 % of the PTV. The mean V95% of the PTV was 98.8 % for RA and 95.9 % for DA (*p* = 0.09). V107 % was 0.15 % for DA vs. 0.01 % for RA; *p* = 0.36. D95 % was superior with RA: 51.3 Gy (97.7 %) vs. 50.0 Gy (95.2 %); *p* = 0.006. Higher conformity index values were obtained with RA: 75.2 vs. 67.9 % (*p* = 0.04). RA led to better homogeneity compared to DA: HI of 0.06 vs. 0.09; *p* = 0.01.

**Table 1:** Summary of dosimetric results for planning target volumes (PTV) on the cohort of 13 patients

Indices	Dynamic arc (DA)	Rapidarc (RA)	*p*-value
D50 % (Gy) (%)	52.5 ± 1.2	52.6 ± 0.7	DA vs RA *p* = 0.54
	100.0 ± 1.7	100.2 ± 0.2	
D95 % (Gy) (%)	50.0 ± 1.2	51.3 ± 0.8	DA vs **RA** *p* = 0.006
	95.2 ± 1.6	97.7 ± 0.9	
D2 % (Gy) (%)	54.3 ± 1.0	53.6 ± 0.8	DA vs **RA** *p* = 0.01
	103.5 ± 1.5	102.1 ± 0.6	
D mean (Gy) (%)	52.2 ± 0.9	52.3 ± 0.7	DA vs RA *p* = 0.17
	99.4 ± 1.2	99.5 ± 1.1	
D98 % (Gy) (%)	49.8 ± 1.9	50.5 ± 0.8	DA vs RA *p* = 0.25
	94.9 ± 2.9	96.2 ± 1.6	
V95 % (%)	95.9 ± 5.5	98.8 ± 1.0	DA vs RA *p* = 0.09
V107 % (%)	0.15 ± 0.5	0.01 ± 0	DA vs RA *p* = 0.36
CI (%)	67.9 ± 8.3	75.2 ± 10.4	DA vs **RA** *p* = 0.04
HI	0.09 ± 0	0.06 ± 0	DA vs **RA** *p* = 0.01

Data presented the mean doses of all patients ± standard deviation. Bold techniques means that this technique is superior for this organ in particular. *Nb* Vx% = minimum x% of prescribed dose received by the volume and Dx% is the minimum dose received by x% of the structure volume, *CI* Conformity Index, *HI* Homogeneity Index

### Organs at risk (Fig. 1b and c)

Table 2 summarizes the results of DVH analyses for OARs. For the ipsilateral cochlea, RA yield the lowest mean dose (Dmean of 25.4 vs. 35.3 Gy, *p* = 0.008). For the hippocampus Dmean and D40% were lower with RA (Dmean of 11.4 vs. 17.5 Gy, *p* = 0.006; D40% of 10.5 vs. 18.1, *p* = 0.005). Also, the mean volume of healthy brain that received a high dose (V90%) was 0.7 % using RA vs. 1.4 % with DA (*p* = 0.05). Inversely, for eyeballs, lenses, pituitary gland (pituitary adenoma excluded) and chiasm, DA was significantly less irradiative compared to RA; both techniques were below the dose constraints.

**Table 2:** Summary of dosimetric results for organs at risk (OAR) on the cohort of 13 patients

Organs		Dynamic arc (DA)	Rapidarc (RA)	*p*-value
Brainstem	D2 % (Gy)	47.4 ± 13.6	47.1 ± 12.0	DA vs RA *p* = 0.81
Spinal cord	D2 % (Gy)	8.9 ± 5.2	1.8 ± 1.9	DA vs **RA** *p* = 10–4
Pituitary gland^a^	D2 % (Gy)	23.7 ± 19	28.6 ± 16.5	**DA** vs RA *p* = 0.01
	D mean (Gy)	17.3 ± 16	22.2 ± 15.4	**DA** vs RA *p* = 0.02
Chiasm	D2 % (Gy)	32.7 ± 22.5	29.3 ± 23.4	DA vs **RA** *p* = 0.01
Hippocampus	D mean (Gy)	16.7 ± 9.2	11.9 ± 5.5	DA vs **RA** *p* = 0.006
	D40 % (Gy)	16.2 ± 10.5	9.9 ± 5.7	DA vs RA *p* = 0.005
Ipsilateral cochlea	D2 % (Gy)	39.4 ± 18.0	33.8 ± 13.8	DA vs **RA** *p* = 0.008
	D mean (Gy)	35.3 ± 18.0	25.4 ± 9.7	DA vs **RA** *p* = 0.008
Controlateral cochlea	D2 % (Gy)	12.5 ± 12.3	17.9 ± 8.4	**DA** vs RA *p* = 0.05
	D mean (Gy)	10.8 ± 11.1	15.8 ± 7.9	**DA** vs RA *p* = 0.04
Cerebellum	D mean (Gy)	8.7 ± 7.0	10.7 ± 5.6	DA vs RA *p* = 0.11
Ipsilateral lens	D2 % (Gy)	1.1 ± 1.5	3.1 ± 2.2	**DA** vs RA *p* = 0.01
	D mean (Gy)	0.9 ± 1.3	2.4 ± 1.9	**DA** vs RA *p* = 0.01
Controlateral lens	D2 % (Gy)	1.4 ± 1.9	3.2 ± 2.1	**DA** vs RA *p* = 0.01
	D mean (Gy)	1.1 ± 1.5	2.5 ± 1.9	**DA** vs RA *p* = 0.01
Ipsilateral optic nerve	D2 % (Gy)	26.8 ± 23.1	26.3 ± 22.5	DA vs RA *p* = 0.83
Controlateral optic nerve	D2 % (Gy)	21.7 ± 19.9	24.0 ± 20.6	DA vs RA *p* = 0.62
Ipsilateral eyeball	D2 % (Gy)	4.1 ± 2.9	9.2 ± 4.5	**DA** vs RA *p* = 10–4
	D mean (Gy)	0.6 ± 0.9	1.4 ± 1.3	DA vs RA *p* = 0.09
Controlateral eyeball	D2 % (Gy)	3.3 ± 3	10.2 ± 4.9	**DA** vs RA *p* = 10–4
	D mean (Gy)	0.2 ± 0.3	1.5 ± 1.4	**DA** vs RA *p* = 0.002
Healthy brain (i-e brain-PTV)	D mean (Gy)	6.7 ± 2.7	5.1 ± 1.8	DA vs **RA** *p* = 0.002
	V100 % (%)	0.4 ± 0.8	0.02 ± 0	DA vs **RA** *p* = 0.02
	V95 % (%)	0.9 ± 1.2	0.3 ± 0.2	DA vs **RA** *p* = 0.03
	V90 % (%)	1.4 ± 1.4	0.7 ± 0.4	DA vs **RA** *p* =0.05
	V80 % (%)	2.2 ± 2.1	1.3 ± 0.7	DA vs RA *p* =0.06

Data presented the mean doses of all patients ± standard deviation. Bold techniques means that this technique is superior for this organ in particular. *Nb* Vx% = minimum x% of prescribed dose received by the volume and Dx% is the minimum dose received by x% of the structure volume. ^a^Pituitary adenomas treatments excluded

### Low-dose irradiation (Fig. 1d)

Table 3 summarizes the results of DVH analyses for low-dose irradiation inside the outer contour. There were no significant differences between mean low-dose volumes of RA and DA: respectively, 184 vs. 166 cm^3^ for V20 Gy (*p* = 0.14) and 1265 vs. 1056 cm^3^ for V5 Gy (*p* = 0.17).

**Table 3:** Summary of dosimetric results for low-dose delivery inside the outer contour on the cohort of 13 patients

Indices	Dynamic arc (DA)	Rapidarc (RA)	*p*-value
Dmean (Gy)	3.9 ± 1.9	3.7 ± 1.7	DA vs RA *p* = 0.41
V30 Gy (cm^3^)	100.5 ± 99.7	92.6 ± 72.9	DA vs RA *p* = 0.49
V20 Gy (cm^3^)	166.1 ± 158.8	184.4 ± 127.0	DA vs RA *p* = 0.14
V10 Gy (cm^3^)	425.1 ± 328.8	476.4 ± 226.4	DA vs RA *p* = 0.09
V5 Gy (cm^3^)	1056.0 ± 569.7	1264.9 ± 1551.6	DA vs RA *p* = 0.17

Data presented the mean doses of all patients ± standard deviation. *Nb* Vx% = minimum x% of prescribed dose received by the volume and Dx% is the minimum dose received by x% of the structure volume

## Discussion

VMAT using RA and non-coplanar DA in FSRT for benign skull-base tumors both provided acceptable dosimetric results. However, RA was more conformal and homogeneous and offered better sparing of OARs. RA also had a faster treatment time.

Target-volume coverage was acceptable for both techniques, with the 95 % isodose covering a higher volume for RA (98.8 vs. 95.9 %; *p* = 0.09). However, this difference was not significant, probably due to the small number of patients in our study. One limit of this comparison is that (especially for DA, which is a forwardly planned technique), the experience of the planner to some extent determines the quality of the plan and thus influences the results of this comparative study.

Few published studies have compared DA and intensity-modulated radiotherapy (IMRT) for intracranial tumors; most have used step-and-shoot IMRT and not VMAT [29–31]. Ding et al. [29] performed a planning study in 15 patients and compared 3D conformal radiotherapy, DA, and step-and-shoot IMRT for FSRT to treat brain tumors. They concluded that both DA and IMRT were suitable in most cases, but that IMRT was best for larger tumors (PTV > 100 cm^3^). Wiggenraad et al. [31] compared step-and-shoot IMRT and DA to treat intracranial tumors at various sites, and of different sizes and shapes, including mostly gliomas and meningioma. They found that both DA and IMRT were acceptable in most cases. However, they found that IMRT offered a statistically better mean CI in concave intracranial tumors. CI quantifies the best adaptation to shape the 95 % isodose envelope to the exact shape of the PTV [28]. We found that RA provided the best target volume conformity (CI of 75.2 vs. 67.9; *p* = 0.04). Similar results have been found when comparing VMAT to DA for different localizations, such as lung and prostate cancers [32–35]. As a result of this better conformity, we found that healthy brain received significantly less high radiation doses with RA (V90% of 0.7 % using RA vs. 1.4 % with DA; *p* = 0.05). This is consistent with the study by Anand et al. [30] who compared RA and DA for recurrent high-grade gliomas that were reirradiated with FSRT. They found a significantly better CI with RA and that RA delivered a significantly lesser dose to previously irradiated high-dose brain volumes, possibly minimizing the risk of radionecrosis.

In our opinion, dose homogeneity is an important aspect of the quality of planning, especially for skull-base tumors where OARs are often located partially inside the PTV. It is important to maintain very good dose homogeneity because intra- and inter-fraction uncertainties may cause serious adverse effects when hotspots occur near these OARs. In our study, the RA plans gave the best homogeneity (HI of 0.06 with RA vs. 0.09 with DA; *p* = 0.01). This result is consistent with most studies that have compared VMAT with DA [32–35].

Another important aspect for FSRT for benign skull-base tumors is to spare critical OARs [36]. We found dosimetric constraints were respected for all OARs using both techniques. However, we found significant dosimetric benefits using RA for the ipsilateral cochlea and the hippocampus. This may be explained by optimization: constraints to these OARs were included in the dose calculation and the inverse planification for RA. In our study, Dmean to the ipsilateral cochlea was significantly lower with RA. Dmean was lower of 10 Gy in average, which can be particularly beneficial in some cases.

Several studies have attempted to relate mean cochlear dose to hearing loss and have reported a significant increase in hearing loss when dose received by the cochlea was >45–50 Gy [36–38]. Sparing of normal tissue is also very crucial for the hippocampus because these benign tumors are associated with a long life expectancy. The association between hippocampal dose and long-term neurocognitive function impairment for benign or low-grade adult brain tumors treated with FSRT has been evaluate by Gondi et al. [20]. In their study, biologically equivalent doses, in 2-Gy fractions (assuming an α/β ratio of 2 Gy) that were >7.3 Gy to 40 % of the bilateral hippocampus, was significantly associated with long-term memory impairment. A phase-II study (RTOG 0933) has confirmed these results in patients treated for brain metastasis [23]. Hippocampal avoidance, defined here as D100 % <9 Gy and Dmax <16 Gy during whole-brain radiotherapy (30 Gy in 10 fractions), was associated with significant memory preservation at 4 months compared to a historical control cohort. Even if dose constraints to the hippocampus are not totally well established, in our study we found that Dmean and D40 % were significantly lower with RA (Dmean of 11.4 vs. 17.5 Gy, *p* = 0.006; D40 % of 10.5 vs. 18.1, *p* = 0.005). In this study, we did not use hard constraints to the hippocampus for inverse planning with RA. It may be possible to lower these doses further with RA using inverse planning and without compromising PTV coverage too much.

Another important issue is comparing the low-dose deliveries of these two techniques. Indeed, some authors support that increasing normal tissue irradiation to low doses might increase the incidence of solid cancers in long-term survivors [39]. In our study, we did not find any significant difference in terms of low-dose volumes between RA and DA. Wiggenraad et al. [31] found similar results; however, they noted that more monitor units were needed with IMRT, even though they did not find a statistically significant difference between IMRT and DA with respect to the volume of irradiated brain tissue.

## Conclusions

FSRT, using coplanar VMAT with Rapidarc®, allowed target-volume coverage that was at least equal to that of non-coplanar multiple dynamic arcs while also offering better sparing of the ipsilateral cochlea and hippocampus. Rapidarc® was also more conformal and homogeneous. Low-dose volumes were similar between both techniques. Treatment time was also reduced with Rapidarc®. In our institution, we now preferentially treat large benign tumors (>3 cm) of the skull base using Rapidarc®.

## Declaration

### Ethics approval and consent to participate

This work did not require any written patient consent. The local ethics committee of Centre Jean Perrin approved this work.

### Consent for publication

Not applicable.

## Abbreviations

CI: conformity index
CTV: clinical target volume
DA: non-coplanar dynamic arcs
DVH: dose–volume histogram
FSRT: fractionated stereotactic radiotherapy
GTV: gross tumor volume
HI: homogeneity index
IMRT: intensity-modulated radiotherapy
OAR: organ at risk
PTV: planning target volume
RA: Rapidarc®
VMAT: volumetric modulated arc therapy
